# Thioetherimide-Modified Cyanate Ester Resin with Better Molding Performance for Glass Fiber Reinforced Composites

**DOI:** 10.3390/polym11091458

**Published:** 2019-09-06

**Authors:** Pengchang Ma, Chuntao Dai, Shaohua Jiang

**Affiliations:** 1 Zhongshan Polytechnic, Zhongshan 528404, China (P.M.) (C.D.); 2 College of Materials Science and Engineering, Nanjing Forestry University, Nanjing 210037, China

**Keywords:** thioetherimide, cyanate resin, mechanical property, dimension stability, moisture absorption, processability

## Abstract

Cyanate ester (CE) resins with higher heat resistance, lower coefficients of thermal expansion (CTEs), and lower water absorption ratios are highly desired in printed circuit boards (PCBs). In this work, a CE was modified by copolymerization with a long-chain thioether bismaleimide (SBMI) to form a thioetherimide-modified CE (SBT). The results indicated that SBT had a wider processing window and better processing properties than a common bismaleimide-modified CE resin (MBMI). After molding with a glass fiber cloth, the composites (GSBT) exhibited moisture adsorption in the range of 1.4%–2.0%, high tensile strength in the range of 311–439 MPa, good mechanical retention of 70%–85% even at 200 °C, and good dimension stability, with coefficients of thermal expansion in the range of 17.3–18.6 (×10^−6^ m/°C). Such GSBT composites with superior properties would be good candidates for PCB applications.

## 1. Introduction

The modern printed circuit board (PCB) industry requires more substrate layers, a higher wiring density, a larger working frequency range, and a smaller insulation thickness. Therefore, novel polymers or polymer composites with higher heat resistance, lower coefficients of thermal expansion (CTE), lower water absorption ratios, and superior dielectric properties are highly required in the PCB industry. Cyanate esters (CEs) are popular thermoset polymeric materials with good processability, excellent moisture and heat resistance, high dimensional stability, good dielectric properties, and much lower cost compared to polyimide resins, which have been widely used in the production of high-frequency PCBs and radar domes [1,2,3,4,5,6]. Although CE resins have excellent comprehensive properties compared to those of epoxy resins, a brittle cured product or a fracture strain of less than 2% can make a CE resin fail to meet many applications, due to its high cross-link density after polymerization. In addition, processing and curing temperatures above 220 °C cause large energy consumption in production. Thus far, many research works have been undertaken to modify CE materials and their composites. To increase the tensile strength of CEs, the chemical structure and phase structure of the curing system have been modified by copolymerization or blending with thermoplastic resins, rubber, rigid particles, thermoset resins, and so on [7,8,9,10,11,12,13]. However, the addition of different types of resin forms networks with different degrees of branching microstructures. These structures can improve the strength of CE resin to a certain extent, but it also decreases the related thermal stabilities.

Bismaleimide copolymers (BT resins) are well-known for their excellent electrical properties, mechanical properties, and heat resistance, and have been widely used in the fields of electronic printing, wave-transparent materials, and aerospace [14,15,16,17,18,19,20]. Bismaleimides (BMIs) have good compatibility with CEs. A homogeneous thermodynamic system can be obtained by fusion, and the cured product has a glass transition temperature of up to 250 °C or higher. Though the BMI has a high heat resistance, its high cross-link density is responsible for the high brittleness of the cured product. The cross-link density of the resin can be reduced by increasing the molecular weight of the BMI pre-polymer, resulting in a resin system with a wider processing window and higher tensile strength. This significantly improves the processability and toughness of the BT resin and provides a shortcut for decreasing the brittleness of BT and CE resins [21,22]. However, the length of the molecular chain of a conventional long-chain BMI resin significantly affects the performance of the BT resin in the molding process, with an increased processing difficulty and a reduced heat resistance in most cases. Using a long-chain BMI with an imide structure avoids the issue of reduced heat resistance but brings about a difficulty in the molding process due to the solubility of the structure and the excessive melt viscosity.

Therefore, in this work, a new type of long-chain thioether BMI (SBMI) resin was synthesized. By controlling its molecular weight and copolymerizing it with a CE, a novel modified cyanate resin with good molding performance and its glass fiber reinforced composites were fabricated. Further comprehensive characterizations were performed on the SBMI and its composites.

## 2. Materials and Methods

### 2.1. Materials

3,3’,4,4’-Diphenylthioether tetrahydrate dianhydride (TDPA) was synthesized via a preparation method reported in the literature [23]. Bisphenol A dicyanate ester (BADCy) and 4,4’-diphenylmethane bismaleimide (BMI) were purchased from Wuqiao Resin Factory (Jiangdu, China). Maleic anhydride was purchased from Shanghai Bayuan Chemical Industry Co., Ltd. (Shanghai, China). 3,3’-Dimethyl-4,4’-diaminodiphenylmethane (DMMDA) was bought from Shanghai Rongbai Biotechnology Co., Ltd. (Shanghai, China). EW170-100 glass fiber cloth was provided by Shenyang Gaote Glass Fiber Products Co., Ltd. (Shenyang, China). This glass fiber cloth is a nonwoven fabric with a surface density of 200 ± 20 g/m^2^. All other reagents were obtained from Tianjin Tiantai Fine Chemical Industry Co., Ltd (Tianjin, China). DMMDA and Maleic anhydride were purified by sublimation in a vacuum prior to use. All other chemicals were used as received unless otherwise specified.

### 2.2. Preparation of the Long-Chain Thioether BMI

**Scheme 1:**
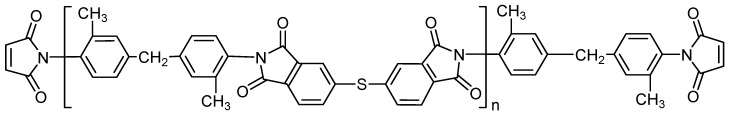
Structures of maleimide-terminated thioetherimide oligomers (1SBMI, 2SBMI).

In a three-necked flask, 2.1664 g (0.01 mol) of diamine DMDDA and 3.0248 g (0.01 mol) of dianhydride TDPA were dissolved in 50 mL of DMAc and then mechanically stirred at room temperature for 12 h. Then, 0.15 g of the capping agent maleic anhydride was added, and the reaction was carried out for 4 h. A quantity of 2.8 g of a catalyst (*n*(acetic anhydride):*n*(triethylamine) = 2:1) was added, stirring was continued for another 12 h, and then the reaction solution was treated with ethanol. After drying under a vacuum, a bismaleimide-terminated thioetherimide oligomer SBMI was obtained. Two oligomers with chain segment numbers (*n*) of 1 and 2 were obtained by adjusting the proportion of reactants (Scheme 1). The bismaleimide resin with *n* = 1 was labeled as 1SBMI, while the bismaleimide resin with *n* = 2 was labeled as 2SBMI.

### 2.3. Preparation of Glass Fiber Cloth Prepreg

The CE was heated at 140–150 °C for 8 h. After being cooled down to room temperature(R.T.), the CE was dissolved in ethylene glycol monomethyl. Then, different amounts of conventional or modified BMI resin (20%, 30%, and 37.5% wt) were added to the corresponding BT resin solutions, respectively. The BT resins obtained by the copolymerization of 1SBMI and CE were labeled as 1SBT_1_, 1SBT_2_, and 1SBT_3_, respectively, while the BT resins obtained from 2SBMI were labeled as 2SBT_1_, 2SBT_2_, and 2SBT_3_, respectively. For comparison, BT resins obtained from common bismaleimide resin (MBMI) were labeled as MBT_1_, MBT_2_, and MBT_3_, respectively [24]. The EW170-100 glass cloth was preimpregnated in the prepreg solutions of modified cyanate resin with a volume ratio of *V*(fiber):*V*(resin) = 3:2. The obtained composites were held for 6 h at R.T. to remove most of the solvent, and then treated at 50–60 °C for 4 h. After the surface was dried, it was cut into 80 mm × 100 mm slices for further use.

### 2.4. Preparation of Composites

**Figure 1:**
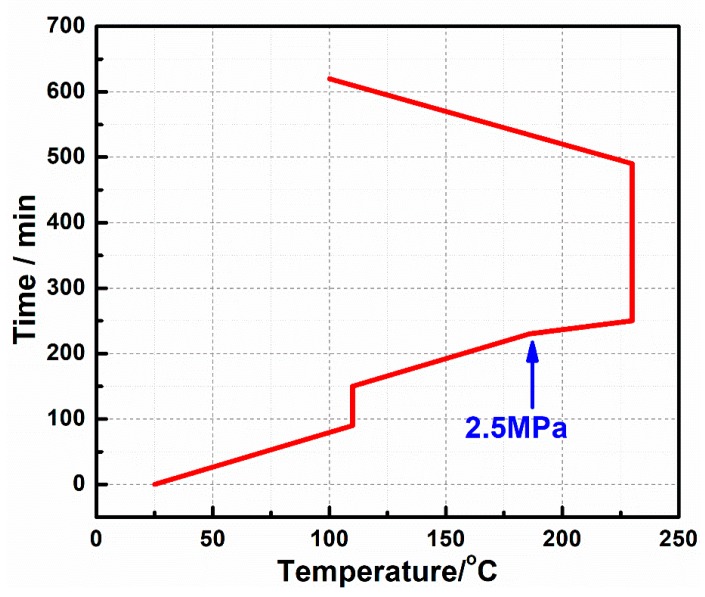
Molding process of composites.

Thirty layers of the above cut slices were laminated in a mold. The thickness of each layer was about 0.14 mm. According to the analysis of the rheological behavior of resins, the molding process is shown in Figure 1. The material was placed on a plate vulcanizing press and maintained at 110 °C for 1 h. After the solvent was completely removed, the temperature was raised to 186 °C at a heating rate of 0.95 °C/min, then a pressure of 2.5 MPa was applied, and then the temperature was continuously raised up to 230 °C at a heating rate of 2.20 °C/min. After 4 h, the temperature naturally dropped to room temperature, and the pressure was released. The obtained glass fiber-reinforced SBT and MBT composites are denoted as GSBT and GMBT, respectively. The volume fraction of glass fiber in the composites was 65.7%. The hot-pressed sheet with a thickness of 4 mm was cut into specimen strips according to GB9341-2000 and GB/T10404-2006 standards, and the mechanical properties were tested at 20 °C and 200 °C, respectively.

### 2.5. Characterization

Rheological measurements of the resins were conducted on an AR2000ex rheometer (TA Instruments, USA) with a round cake of 25 mm diameter and 1 mm thickness, at a heating rate of 4 °C/min under a flow mode, with 5% strain and an angular frequency of 10 rad/s. Fourier transform infrared spectroscopy (FT-IR) was performed using a Bruker VERTEX 70 spectrometer (Germany). Thermogravimetric analysis (TGA) was carried out on a TGA Q50 (TA Instruments, USA). The samples were heated from 60 to 800 °C at a heating rate of 10 °C/min and a nitrogen flow rate of 60 mL/min. Tensile and flexural properties were tested by an Instron material testing system (Model 5982, Shanghai, China) with a sample size of 80 mm × 10 mm × 4 mm, at a constant displacement rate of 2.0 mm/min, according to GB9341-2000 (ISO 527-4:1997, IDT) and GB/T10404-2006 (ISO 178:1993, IDT) standards. During tensile tests, the span length and gauge length were 60 ± 0.5 mm and 50 ± 0.5 mm, respectively. The span length for the flexural test was 40 ± 0.5 mm. The linear thermal expansion coefficients (CTEs) of the composites were measured by a thermomechanical analyzer (TMA, Q400, TA Instruments, USA) with a sample size of 5 mm × 15 mm, at a heating rate of 10 °C/min. The moisture absorption rate of the composites was measured according to standard ASTM D5229. The measurements for tensile, flexural, and moisture absorption (hygroscopicity) properties were repeated five times for each sample.

## 3. Results and Discussion

### 3.1. Infrared Spectrum of Synthetic Resin

**Figure 2:**
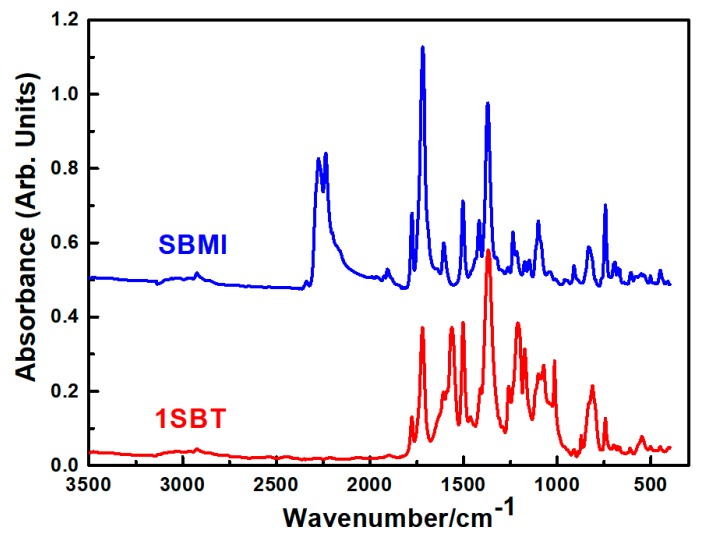
Fourier transform infrared (FT-IR) spectra of 1SBMI and 1SBT resins.

Figure 2 shows the FT-IR spectra of the thioether bismaleimide resin (1SBMI) and 1SBT resin. As can be seen from Figure 2, the peaks at 1720 cm^−1^ and 1780 cm^−1^ [25,26,27] could be attributed to the stretching frequency of imide in the SBMI resin. The stretching frequency of the C–S–C bond was at 1220 to 1280 cm^−1^. The characteristic absorption peak of the C–N bond on the imide ring and the carbon–carbon double bond were located at 1371 cm^−1^ and 833 cm^−1^ [27,28], respectively. The characteristic absorption peak for cyanate was at 2270 cm^−1^. When the curing reaction time was prolonged and the temperature was increased, the carbon–carbon double bond characteristic absorption peak of 1SBT resin at 2270 cm^−1^ completely disappeared, which indicated that 1SBT resin was completely cured under this condition. Meanwhile, the absorption peak of the triazine ring at 1362 cm^−1^ also proved that the CE underwent a cross-linking reaction.

### 3.2. Rheological Properties of Resins

The investigation on the viscosity value at different temperatures is important for the determination of the curing temperature of a resin and the process parameters of a composite. The viscosity of a resin depends on temperature and holding time. When the temperature rises, the kinetic energy of molecular thermal motion increases, lowering the viscosity of the resin. On the other hand, thermal motion can promote the curing reaction of the resin. At a certain temperature, as the curing time increases, both the molecular weight and viscosity increased.

**Figure 3:**
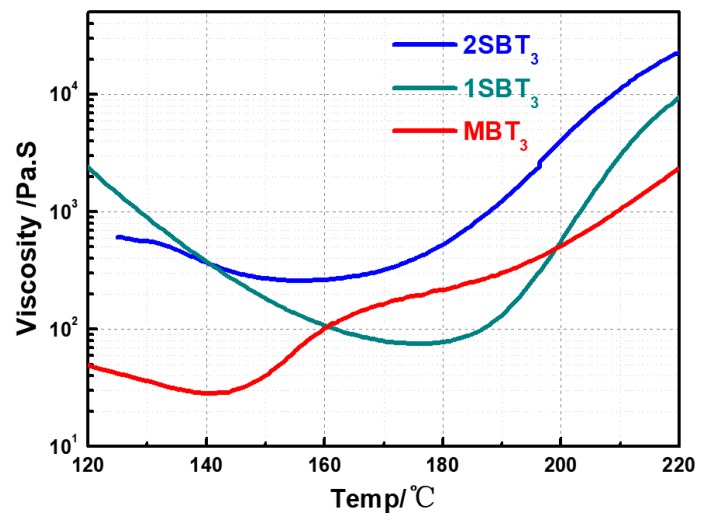
Rheology curves of 2SBT_3_, 1SBT_3_, and MBT_3_ resins.

Figure 3 shows the temperature-dependent viscosity results of SBT and MBT. The viscosity of the resin decreased rapidly at first when the temperature rose, which was due to the melting of the resin crystal. The minimum melting viscosities of MBT_3_, 1SBT_3_, and 2SBT_3_ resins were 28.5 Pa·s [24], 75.1 Pa·s, and 258.3 Pa·s, respectively. The molecular weight of the 1SBMI resin was less than that of the 2SBMI resin, so the melt viscosity of the 1SBT resin was lower than that of the 2SBT resin. However, the viscosity of the MBT_3_ resin increased sharply above 140 °C. In this case, the solvent would be difficult to remove, and premature pressurization resulted in the higher porosity of the composite. Meanwhile, the overflow loss of the MBT_3_ resin resulted in a narrower processing window, which made it very difficult to select an appropriate pressurization time. When the temperature was raised to 160 °C, its melt viscosity increased by an order of magnitude, reaching more than 100 Pa·s. However, if the pressurization was not conducted before the MBT_3_ resin had been cured, this led to a non-uniform interface with the fibers. Thus, the narrow temperature window increased the difficulty of processing and further influenced the properties of the MBT_3_ resin.

As shown in Figure 3, when the temperature was in the range 140–180 °C, the 1SBT resin sustained a low viscosity, relative stability, good fluidity, and infiltrating performance. The 2SBT resin, at 160–200 °C, benefited from the introduction of a thioether bond into the bismaleimide resin. However, after the temperature exceeded 200 °C, the viscosity of the system started to rise sharply, caused by the double bond in the bismaleimide resin and the cross-link reaction of the cyanate resin. Compared with the MBT, the 1SBT and 2SBT resins exhibited a wider processing window and better processing properties. The processing technique of the composite material, as shown in Figure 1 above, was established based on the rheological behavior of the resin.

### 3.3. Thermogravimetric Analysis

**Figure 4:**
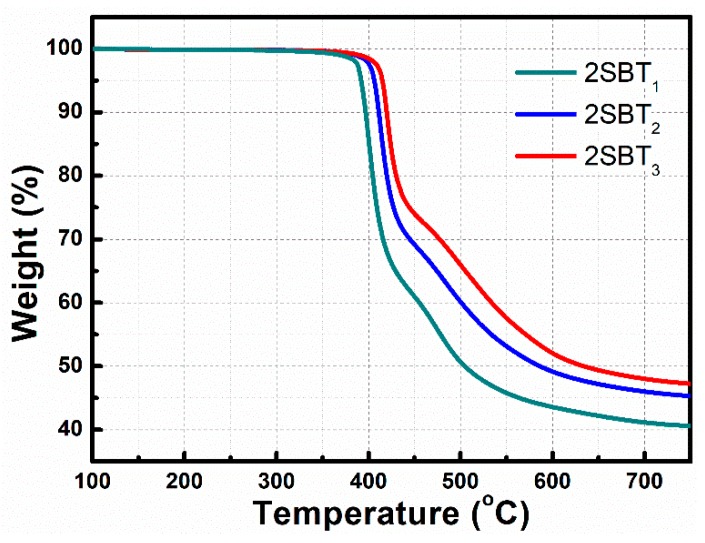
Thermogravimetric analysis (TGA) curves of 2SBT1, 2SBT2, and 2SBT3.

Basically, it was found that the modification of the CE resin showed a tiny effect on the 5% thermal weight loss temperature (*T*_5%_, Table 1). It was shown that the value of *T*_5%_ does not depend on the conventional bismaleimide-modified resin. When the content of BMI resin reached 37.5%, the *T*_5%_ of 1SBT_3_ and 2SBT_3_ (Figure 4) resins were 414 °C and 415 °C, respectively, only slightly higher than that of the pure CE (410 °C) [26]. This might be due to the lower molecular weight of the conventional BMI and the higher cross-link density after curing. So, the thermal stability did not show any significant change, even when it was introduced to the CE resin. On the other hand, although the molecular weight of the SBMI-type BMI was higher than that of the ordinary BMI, the cross-link density was lower than that of conventional BMI resin. Both factors had little effect on the thermal stability of the resin, but along with the introduction of aromatic imide with higher thermal stability in the molecular structure, the obtained modified CE resin as well as its composite material exhibited excellent thermal stability. However, 1SBMI and 2SBMI CE resins modified with the same content showed no great differences in the *T*_5%_ results, because even though the number of chain segments in the two resins were different, their molecular weights were similar, and cross-link reactions occurred at high temperatures, leading to the consistency of thermal stability.

### 3.4. Hygroscopicity Analysis

The hygroscopicity of the three BT composites is shown in Table 2. With increasing MBMI content, the hygroscopicity of MBT gradually increased from 2.8% to 3.7%. From the previous results, the moisture uptake of MBMI resin is 4.5%, while that of CE resin is 2.5% [24,29,30]. The content and structure of the two components determined the moisture absorption ratio of the composite GMBT. With increasing SBMI content, the moisture absorption ratio of the composite GSBT decreased gradually to less than 2.5%. Profiting from the hydrophobic methyl groups and sulfur bonds of SBMI, its own moisture absorption ratio was lower than 1.5%, which made the CE resin lower than 2.5% [24,31,32]. Furthermore, the interpenetrating network structure of SBT after curing and molding also contributed to the decrease in the moisture absorption ratio. However, with the same content of SBMI, the moisture uptake of the composite 2SBT was higher than that of G1SBT. For example, the moisture absorption ratio of G2SBT_3_ was 1.5%, which was higher than the 1.4% of G1SBT_3_, due to the lower proportion of hydrophobic methyl and sulfur bonds in the molecule, correspondingly reduced by the increase in the molecular weight of the composite.

### 3.5. Mechanical Properties of Composites

**Figure 5:**
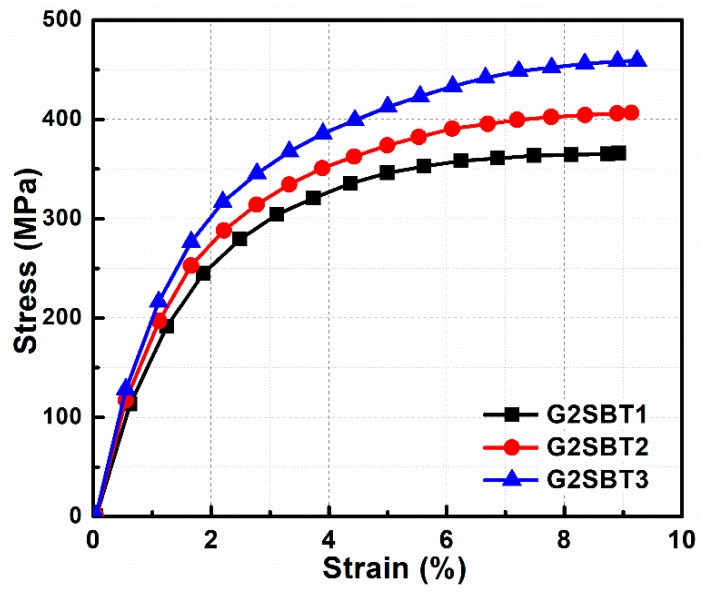
Typical stress-strain curves of G2SBT1, G2SBT2, and G2SBT3.

The glass cloth prepreg prepared from modified CE resin had good molding processability. The pressurization point was easily handled, and the surface of the prepared plate was flat and smooth without defects or warpage. The mechanical properties of the EW170-100 glass cloth reinforced composites at room temperature and elevated temperature are listed in Table 3 and Table 4. The data showed that the tensile strength, flexural strength, and elongation at break of the composite material increased with the increase in the content of the BMI resin. When the content of BMI reached 37.5%, the mechanical properties were greatly improved. Therefore, a comparison point was selected for the mechanical properties of the composites with a BMI content of 37.5%. At 20 °C room temperature, the tensile strength of G1SBT3 was 431 MPa (Figure 5), and that of G2SBT3 was 439 MPa. However, the tensile strength of the conventional BT resin matrix glass cloth reinforced composite was 428 MPa when the content of BMI was controlled [24]. Meanwhile, the flexural strengths of G1SBT_3_ and G2SBT_3_ exceeded 600 MPa, much higher than the 464 MPa of the composite GMBT_3_ [21,24]. When the test temperature reached 200 °C, the tensile strengths of the composite materials G1SBT_3_ and G2SBT_3_ were more than 300 MPa, higher than that of the conventional BT resin matrix composite GMBT_3_ (290 MPa) [24]. The flexural strengths of G1SBT_3_ and G2SBT_3_ were 278 MPa and 307 MPa, respectively, while that of GMBT_3_ was only 264.4 MPa [24], making the former two 5% and 16% higher than GMBT_3_, respectively.

The data in the table also illustrate that the elongation at break of the G2SBT composite was the highest at the same content of MBI or SBMI. It was considered that this low ductility of the resin after curing was caused by the low molecular weight and the high cross-link density of BMI. In addition, the G2SBMI resin had more chains than the G1SBMI resin, and the lengths of both molecular strands were longer than that of the BMI resin, decreasing the cross-link density of the resin after curing to a certain extent, thereby improving the ductility of the material.

### 3.6. Dimensional Stability

The CTEs of the three BT resin composites after curing are listed in Table 5. It can be seen that with the increase in BMI content, the CTE of the GMBT gradually decreased from 17.1 ppm/°C to 15.7 ppm/°C, which was related to the rigid structure of MBI: The more rigid the structure, the lower the CTE. Also, it was related to the cross-link density of the cured resin: The higher the cross-link density, the stronger the rigidity and the lower the CTE. For the SBMI with the same number of chain segments, the CTE of GSBT increased gradually with the increase in SBMI content, because the longer molecular strand of SBMI caused the lower cross-link density of the resin after curing, reducing the rigidity of the molecular strands after curing. Although the SBMI molecular chain contained a methyl rigid structural group, it also contained a flexible ether bond in the molecular chain, and the combined effect of the two led to the final effect on the flexibility of the molecular chain. Similarly, the chain length of 2SBMI was longer than that of 1SBMI, and both were longer than that of MBI. After curing, the cross-link density of the resin was correspondingly lowered, and the rigidity was also reduced. Therefore, given the same content of BMI resin, the CTE of G2SBT was correspondingly higher than that of G1SBT, and both were higher than the GMBH with the same BMI content. However, the increase is not significant enough to affect its application in PCBs.

## 4. Conclusions

We prepared a long-chain thioether bismaleimide, which was used as a modifier to a CE resin. The long-chain SBMI was demonstrated to have a higher molecular weight, higher hydrophobicity, and better thermal stability than the conventional BMI. It was found to offer good processability, and its cured materials and composites had better heat resistance and mechanical properties, with lower moisture absorption and linear expansion coefficients. The modified resin 1SBT3 using an SBMI content of 37.5% had a 5% thermal weight loss temperature as high as 414 °C. The tensile strengths of the composite material at room temperature and at 200 °C were 431.2 MPa and 331.0 MPa, respectively, and the flexural strengths were 631.5 MPa and 278.4 MPa, respectively. The X-Y axis CTE of the composite was 18.1×10^−6^ m/°C, and the hygroscopic coefficient was about 1.4%. All the above data indicated that CE resin matrix composite materials modified by long-chain SBMI have excellent comprehensive performance with promising applications for PCBs.

## Figures and Tables

**Table 1:** Thermal properties of three kinds of cured bismaleimide copolymers (BT) resin.

Code	*T*_5__%_ (°C)	Code	*T*_5__%_ (°C)	Code	*T*_5__%_ (°C)
1SBT_1_	395	2SBT_1_	392	MBT_1_	404
1SBT_2_	405	2SBT_2_	406	MBT_2_	407
1SBT_3_	414	2SBT_3_	415	MBT_3_	413

**Table 2:** Moisture absorption ratios of MBT, 1SBT, and 2SBT composites (24 h).

Entry	GMBT_1_	GMBT_2_	GMBT_3_	G1SBT_1_	G1SBT_2_	G1SBT_3_	G2SBT_1_	G2SBT_2_	G2SBT_3_
Moisture absorption (%)	2.8 ± 0.2	3.5 ± 0.4	3.7 ± 0.3	1.9 ± 0.2	1.5 ± 0.1	1.4 ± 0.1	2.0 ± 0.2	1.6 ± 0.1	1.5 ± 0.1

**Table 3:** Mechanical properties of the G1SBT composites.

Code	Test Temperature (°C)	Tensile Strength (MPa)	Retention Rate (%)	Elongation at Break (%)	Flexural Strength (MPa)	Retention Rate (%)
G1SBT_1_	20	366 ± 35	85	8.2 ± 0.7	576 ± 56	47
	200	311 ± 30		10.2 ± 1.2	270 ± 38	
G1SBT_2_	20	384 ± 46	84	8.4 ± 0.9	604 ± 59	46
	200	323 ± 31		10.4 ± 1.0	278 ± 26	
G1SBT_3_	20	431 ± 55	70	8.7 ± 0.4	614 ± 72	45
	200	331 ± 42		10.8 ± 0.5	278 ± 16	

**Table 4:** Mechanical properties of the G2SBT composites.

Code	Test Temperature (°C)	Tensile Strength (MPa)	Retention Rate (%)	Elongation at Break (%)	Flexural Strength (MPa)	Retention Rate (%)
G2SBT_1_	20	373 ± 42	85	8.9 ± 0.9	585 ± 67	48
	200	317 ± 35		11.2 ± 1.3	279 ± 29	
G2SBT_2_	20	419 ± 43	84	9.1 ± 0.7	647 ± 53	47
	200	352 ± 27		12.4 ± 1. 4	307 ± 42	
G2SBT_3_	20	439 ± 32	71	9.2 ± 0.6	657 ± 68	46
	200	382 ± 36		12.5 ± 1.5	307 ± 32	

**Table 5:** X-Y axis coefficients of thermal expansion (CTEs) for MBT, 1SBT, and 2SBT composites (100–250 °C).

Entry	GMBT_1_	GMBT_2_	GMBT_3_	G1SBT_1_	G1SBT_2_	G1SBT_3_	G2SBT_1_	G2SBT_2_	G2SBT_3_
CTE (×10^−6^m/°C)	17.1	16.6	15.7	17.3	17.8	18.1	17.5	18.3	18.6
